# Diaqua­bis­(5-carb­oxy-1*H*-imidazole-4-carboxyl­ato-κ^2^ *N* ^3^,*O* ^4^)iron(II)

**DOI:** 10.1107/S1600536811024779

**Published:** 2011-06-30

**Authors:** Chao-Jun Du, Xing-Hua Song, Li-Sheng Wang, Chao-Ling Du

**Affiliations:** a Department of Chemical and Biochemical Engineering, Nanyang Institute of Technology, 473004 Nanyang, Henan, People’s Republic of China; b School of Chemical Engineering and Environment, Beijing Institute of Technology, 100081 Beijing, People’s Republic of China; c College of Science, Nanjing University of Aeronautics and Astronautics, Nanjing 211100, People’s Republic of China

## Abstract

In the title compound, [Fe(C_5_H_3_N_2_O_4_)_2_(H_2_O)_2_], the Fe^II^ ion lies on an inversion centre and is coordinated by two N and two O atoms from two 5-carb­oxy-1*H*-imidazole-4-carboxyl­ate ligands and two water mol­ecules in a distorted octa­hedral geometry. An intra­molecular O—H⋯O hydrogen bond occurs. In the crystal, inter­molecular N—H⋯O and O—H⋯O hydrogen bonds form a three-dimensional network, which consolidates the packing.

## Related literature

For the diversity of coordination architectures of the metal atom in complexes with 4,5-dicarb­oxy­imidazole, see: Shimizu *et al.* (2004 ▶); Fang & Zhang (2006 ▶). For the closely related crystal structures of the Zn, Mg and Cd complexes, see: Ma *et al.* (2003 ▶), Liu *et al.* (2004 ▶) and Zhang *et al.* (2004 ▶), respectively.
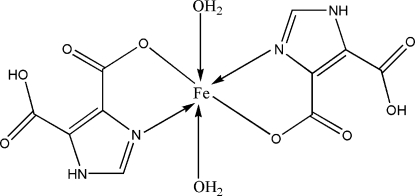


## Experimental

### 

#### Crystal data

[Fe(C_5_H_3_N_2_O_4_)_2_(H_2_O)_2_]
*M* *_r_* = 402.07
Monoclinic, 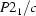

*a* = 5.0676 (9) Å
*b* = 22.769 (4) Å
*c* = 6.6725 (9) Å
β = 113.733 (10)°
*V* = 704.8 (2) Å^3^
*Z* = 2
Mo *K*α radiation
μ = 1.14 mm^−1^
*T* = 298 K
0.32 × 0.28 × 0.25 mm

#### Data collection

Bruker SMART APEXII CCD area-detector diffractometer
Absorption correction: multi-scan (*SADABS*; Bruker, 2005 ▶) *T*_min_ = 0.712, *T*_max_ = 0.764
2872 measured reflections
1240 independent reflections
976 reflections with *I* > 2σ(*I*)
*R*_int_ = 0.027

#### Refinement

*R*[*F* ^2^ > 2σ(*F* ^2^)] = 0.034
*wR*(*F* ^2^) = 0.080
*S* = 1.05
1240 reflections
116 parameters
H-atom parameters constrained
Δρ_max_ = 0.25 e Å^−3^
Δρ_min_ = −0.25 e Å^−3^

### 

Data collection: *APEX2* (Bruker, 2005 ▶); cell refinement: *SAINT* (Bruker, 2005 ▶); data reduction: *SAINT*; program(s) used to solve structure: *SHELXTL* (Sheldrick, 2008 ▶); program(s) used to refine structure: *SHELXTL*; molecular graphics: *SHELXTL*; software used to prepare material for publication: *SHELXTL*.

## Supplementary Material

Crystal structure: contains datablock(s) global, I. DOI: 10.1107/S1600536811024779/cv5111sup1.cif

Structure factors: contains datablock(s) I. DOI: 10.1107/S1600536811024779/cv5111Isup2.hkl

Additional supplementary materials:  crystallographic information; 3D view; checkCIF report

## Figures and Tables

**Table 1:** Hydrogen-bond geometry (Å, °)

*D*—H⋯*A*	*D*—H	H⋯*A*	*D*⋯*A*	*D*—H⋯*A*
N2—H2⋯O3^i^	0.86	2.05	2.897 (3)	169
O3—H3⋯O2	0.82	1.74	2.525 (3)	160
O1*W*—H1*W*⋯O2^ii^	0.85	1.94	2.744 (3)	157
O1*W*—H2*W*⋯O1^iii^	0.85	1.92	2.710 (3)	155

Symmetry codes: (i) 
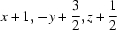
; (ii) 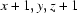; (iii) 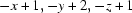.

**Fig. 1.**
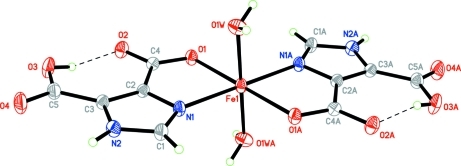
The molecular structure of (I), with displacement ellipsoids drawn at the 40% probability level [symmetry code: (A) 2 - x, 2 - y, 1 - z].

## supplementary crystallographic information

### Comment

In recent years, the construction of metal complexes based on 1*H*-imidazole-4,5-dicarboxylic acid ligand has been investigated in terms of their intriguing topologies. The diversity of coordination architecture of metal 4,5-dicarboxyimidazole has been described by Shimizu *et al.* (2004) and Fang *et al.* (2006). In order to search for new metal complexes based on 1*H*-imidazole-4,5-dicarboxylic acid ligand, the title complex (I) has been synthesized and its crystal determined.

The crystal structure of (I) is isostructural with the previously reported Zn (Ma *et al.*, 2003), Mg (Zhang *et al.*, 2004) and Cd (Liu *et al.*, 2004) 4,5-dicarboxyimidazole complexes. In the four isostructural complexes, all metal ions lie on an inversion centre being coordinated by two *N,O*-bidentate 1*H*-imidazole-4,5-dicarboxylate monoanionic ligands and two water molecules in a distorted octahedral geometry.

In the crystal structure of (I), intermolecular N—H···O and O—H···O hydrogen bonds (Table 1) form three-dimensional hydrogen-bonding network, which consolidate the crystal packing.

### Experimental

A mixture of FeSO_4_^.^7H_2_O (0.10 mmol), 1*H*-imidazole-4,5-dicarboxylic acid (0.10 mmol), Et_3_N (0.1 ml), EtOH (2 ml) and H_2_O (2 ml) was sealed in a 10 ml Tefon-lined stainless-steel reactor and then heated to 393 K for 48 h under autogenous pressure, and then slowly cooled to room temperature at a rate of 5 K/h. Pale-yellow block crystals of the title complex were isolated, washed with distilled water, and dried in air (yield: 48%).

### Refinement

H atoms attached to C and N atoms were placed in calculated positions (C—H = 0.93 Å, N—H = 0.86 Å) and refined as riding atoms and with *U*_iso_(H) = 1.2 *U*_eq_(C, N),respectively. The carboxy and water H atoms were located in a difference map, but placed in idealized positions (O—H = 0.82, 0.85 Å), and refined as riding with *U*_iso_(H) = 1.5 *U*_eq_(O).

### Crystal data

**Table:**

[Fe(C_5_H_3_N_2_O_4_)_2_(H_2_O)_2_]	*F*(000) = 408
*M**_r_* = 402.07	*D*_x_ = 1.895 Mg m^−^^3^
Monoclinic, *P*2_1_/*c*	Mo *K*α radiation, λ = 0.71073 Å
Hall symbol: -P 2ybc	Cell parameters from 1102 reflections
*a* = 5.0676 (9) Å	θ = 3.5–23.7°
*b* = 22.769 (4) Å	µ = 1.14 mm^−^^1^
*c* = 6.6725 (9) Å	*T* = 298 K
β = 113.733 (10)°	Block, pale-yellow
*V* = 704.8 (2) Å^3^	0.32 × 0.28 × 0.25 mm
*Z* = 2	

### Data collection

**Table:**

Bruker SMART APEXII CCD area-detector diffractometer	1240 independent reflections
Radiation source: fine-focus sealed tube	976 reflections with *I* > 2σ(*I*)
graphite	*R*_int_ = 0.027
φ and ω scans	θ_max_ = 25.2°, θ_min_ = 3.5°
Absorption correction: multi-scan (*SADABS*; Bruker, 2005)	*h* = −6→5
*T*_min_ = 0.712, *T*_max_ = 0.764	*k* = −26→26
2872 measured reflections	*l* = −4→7

### Refinement

**Table:**

Refinement on *F*^2^	Primary atom site location: structure-invariant direct methods
Least-squares matrix: full	Secondary atom site location: difference Fourier map
*R*[*F*^2^ > 2σ(*F*^2^)] = 0.034	Hydrogen site location: inferred from neighbouring sites
*wR*(*F*^2^) = 0.080	H-atom parameters constrained
*S* = 1.05	*w* = 1/[σ^2^(*F*_o_^2^) + (0.0375*P*)^2^ + 0.0621*P*] where *P* = (*F*_o_^2^ + 2*F*_c_^2^)/3
1240 reflections	(Δ/σ)_max_ < 0.001
116 parameters	Δρ_max_ = 0.25 e Å^−^^3^
0 restraints	Δρ_min_ = −0.25 e Å^−^^3^

### Special details

**Table:**

Geometry. All e.s.d.'s (except the e.s.d. in the dihedral angle between two l.s. planes) are estimated using the full covariance matrix. The cell e.s.d.'s are taken into account individually in the estimation of e.s.d.'s in distances, angles and torsion angles; correlations between e.s.d.'s in cell parameters are only used when they are defined by crystal symmetry. An approximate (isotropic) treatment of cell e.s.d.'s is used for estimating e.s.d.'s involving l.s. planes.
Refinement. Refinement of *F*^2^ against ALL reflections. The weighted *R*-factor *wR* and goodness of fit *S* are based on *F*^2^, conventional *R*-factors *R* are based on *F*, with *F* set to zero for negative *F*^2^. The threshold expression of *F*^2^ > σ(*F*^2^) is used only for calculating *R*-factors(gt) *etc*. and is not relevant to the choice of reflections for refinement. *R*-factors based on *F*^2^ are statistically about twice as large as those based on *F*, and *R*- factors based on ALL data will be even larger.

### Fractional atomic coordinates and isotropic or equivalent isotropic displacement parameters (Å^2^)

**Table:**

	*x*	*y*	*z*	*U*_iso_*/*U*_eq_	
C1	1.2587 (6)	0.86826 (13)	0.4808 (4)	0.0322 (7)	
H1	1.4476	0.8718	0.5837	0.039*	
C2	0.8201 (6)	0.88774 (11)	0.2661 (4)	0.0265 (6)	
C3	0.8639 (6)	0.82984 (12)	0.2373 (4)	0.0279 (6)	
C4	0.5611 (5)	0.92523 (12)	0.1742 (4)	0.0268 (6)	
C5	0.6789 (7)	0.78182 (14)	0.1066 (5)	0.0369 (7)	
Fe1	1.0000	1.0000	0.5000	0.0278 (2)	
N1	1.0693 (5)	0.91158 (10)	0.4203 (3)	0.0289 (6)	
N2	1.1435 (5)	0.81868 (10)	0.3749 (4)	0.0335 (6)	
H2	1.2302	0.7855	0.3906	0.040*	
O1	0.5823 (4)	0.97682 (8)	0.2452 (3)	0.0313 (5)	
O2	0.3337 (4)	0.90334 (9)	0.0295 (3)	0.0370 (5)	
O3	0.4133 (4)	0.79590 (9)	−0.0221 (3)	0.0462 (6)	
H3	0.3975	0.8318	−0.0301	0.069*	
O4	0.7646 (5)	0.73195 (9)	0.1211 (4)	0.0515 (6)	
O1W	0.8685 (4)	0.96305 (9)	0.7355 (3)	0.0392 (5)	
H1W	1.0038	0.9508	0.8520	0.059*	
H2W	0.7663	0.9851	0.7785	0.059*	

### Atomic displacement parameters (Å^2^)

**Table:**

	*U*^11^	*U*^22^	*U*^33^	*U*^12^	*U*^13^	*U*^23^
C1	0.0267 (15)	0.0259 (17)	0.0363 (16)	0.0013 (13)	0.0045 (12)	−0.0015 (13)
C2	0.0257 (15)	0.0223 (16)	0.0300 (14)	−0.0002 (12)	0.0098 (12)	0.0011 (12)
C3	0.0274 (16)	0.0224 (16)	0.0320 (15)	0.0013 (13)	0.0101 (12)	0.0011 (12)
C4	0.0259 (16)	0.0258 (18)	0.0268 (14)	0.0010 (12)	0.0088 (12)	0.0022 (12)
C5	0.0379 (18)	0.0283 (18)	0.0430 (18)	−0.0011 (15)	0.0144 (14)	−0.0057 (14)
Fe1	0.0267 (3)	0.0203 (3)	0.0314 (3)	0.0024 (3)	0.0067 (2)	−0.0026 (2)
N1	0.0271 (13)	0.0217 (13)	0.0331 (12)	0.0008 (11)	0.0073 (10)	−0.0030 (10)
N2	0.0316 (14)	0.0218 (14)	0.0432 (14)	0.0079 (11)	0.0110 (11)	0.0020 (11)
O1	0.0282 (11)	0.0220 (11)	0.0375 (11)	0.0047 (9)	0.0068 (9)	−0.0019 (9)
O2	0.0277 (11)	0.0317 (12)	0.0402 (11)	0.0007 (10)	0.0018 (9)	0.0000 (9)
O3	0.0367 (12)	0.0286 (13)	0.0570 (14)	−0.0070 (10)	0.0019 (10)	−0.0073 (11)
O4	0.0512 (15)	0.0258 (13)	0.0703 (16)	−0.0008 (11)	0.0169 (12)	−0.0131 (11)
O1W	0.0346 (12)	0.0437 (14)	0.0385 (11)	0.0168 (10)	0.0140 (9)	0.0092 (10)

### Geometric parameters (Å, °)

**Table:**

C1—N1	1.321 (3)	C5—O3	1.312 (3)
C1—N2	1.335 (3)	Fe1—O1W^i^	2.1128 (19)
C1—H1	0.9300	Fe1—O1W	2.1128 (19)
C2—C3	1.363 (4)	Fe1—N1	2.147 (2)
C2—N1	1.379 (3)	Fe1—N1^i^	2.147 (2)
C2—C4	1.476 (4)	Fe1—O1	2.1801 (18)
C3—N2	1.367 (3)	Fe1—O1^i^	2.1801 (18)
C3—C5	1.475 (4)	N2—H2	0.8600
C4—O1	1.255 (3)	O3—H3	0.8200
C4—O2	1.270 (3)	O1W—H1W	0.8498
C5—O4	1.206 (3)	O1W—H2W	0.8499
			
N1—C1—N2	111.1 (2)	O1W^i^—Fe1—O1	90.82 (7)
N1—C1—H1	124.4	O1W—Fe1—O1	89.18 (7)
N2—C1—H1	124.4	N1—Fe1—O1	77.53 (8)
C3—C2—N1	109.5 (2)	N1^i^—Fe1—O1	102.47 (8)
C3—C2—C4	132.0 (2)	O1W^i^—Fe1—O1^i^	89.18 (7)
N1—C2—C4	118.4 (2)	O1W—Fe1—O1^i^	90.82 (7)
C2—C3—N2	105.6 (2)	N1—Fe1—O1^i^	102.47 (8)
C2—C3—C5	134.4 (2)	N1^i^—Fe1—O1^i^	77.53 (7)
N2—C3—C5	120.0 (2)	O1—Fe1—O1^i^	179.999 (1)
O1—C4—O2	124.6 (2)	C1—N1—C2	105.6 (2)
O1—C4—C2	117.2 (2)	C1—N1—Fe1	142.71 (18)
O2—C4—C2	118.2 (2)	C2—N1—Fe1	111.22 (17)
O4—C5—O3	121.6 (3)	C1—N2—C3	108.2 (2)
O4—C5—C3	121.9 (3)	C1—N2—H2	125.9
O3—C5—C3	116.5 (3)	C3—N2—H2	125.9
O1W^i^—Fe1—O1W	179.998 (1)	C4—O1—Fe1	115.45 (16)
O1W^i^—Fe1—N1	93.27 (8)	C5—O3—H3	109.5
O1W—Fe1—N1	86.73 (8)	Fe1—O1W—H1W	115.5
O1W^i^—Fe1—N1^i^	86.73 (8)	Fe1—O1W—H2W	115.7
O1W—Fe1—N1^i^	93.27 (8)	H1W—O1W—H2W	105.2
N1—Fe1—N1^i^	179.999 (1)		
			
N1—C2—C3—N2	−0.4 (3)	O1W^i^—Fe1—N1—C1	96.8 (3)
C4—C2—C3—N2	−176.4 (3)	O1W—Fe1—N1—C1	−83.2 (3)
N1—C2—C3—C5	176.3 (3)	O1—Fe1—N1—C1	−173.1 (3)
C4—C2—C3—C5	0.3 (5)	O1^i^—Fe1—N1—C1	6.9 (3)
C3—C2—C4—O1	174.6 (3)	O1W^i^—Fe1—N1—C2	−93.03 (18)
N1—C2—C4—O1	−1.1 (4)	O1W—Fe1—N1—C2	86.97 (18)
C3—C2—C4—O2	−5.5 (4)	O1—Fe1—N1—C2	−2.91 (16)
N1—C2—C4—O2	178.8 (2)	O1^i^—Fe1—N1—C2	177.09 (16)
C2—C3—C5—O4	−174.3 (3)	N1—C1—N2—C3	0.1 (3)
N2—C3—C5—O4	1.9 (5)	C2—C3—N2—C1	0.2 (3)
C2—C3—C5—O3	3.9 (5)	C5—C3—N2—C1	−177.1 (3)
N2—C3—C5—O3	−179.8 (3)	O2—C4—O1—Fe1	178.5 (2)
N2—C1—N1—C2	−0.3 (3)	C2—C4—O1—Fe1	−1.6 (3)
N2—C1—N1—Fe1	170.2 (2)	O1W^i^—Fe1—O1—C4	95.72 (19)
C3—C2—N1—C1	0.4 (3)	O1W—Fe1—O1—C4	−84.28 (19)
C4—C2—N1—C1	177.0 (2)	N1—Fe1—O1—C4	2.55 (18)
C3—C2—N1—Fe1	−173.45 (18)	N1^i^—Fe1—O1—C4	−177.45 (18)
C4—C2—N1—Fe1	3.2 (3)		

Symmetry codes: (i) −*x*+2, −*y*+2, −*z*+1.

### Hydrogen-bond geometry (Å, °)

**Table:**

*D*—H···*A*	*D*—H	H···*A*	*D*···*A*	*D*—H···*A*
N2—H2···O3^ii^	0.86	2.05	2.897 (3)	169
O3—H3···O2	0.82	1.74	2.525 (3)	160
O1W—H1W···O2^iii^	0.85	1.94	2.744 (3)	157
O1W—H2W···O1^iv^	0.85	1.92	2.710 (3)	155

Symmetry codes: (ii) *x*+1, −*y*+3/2, *z*+1/2; (iii) *x*+1, *y*, *z*+1; (iv) −*x*+1, −*y*+2, −*z*+1.
